# The *LRP6* rs2302685 polymorphism is associated with increased risk of myocardial infarction

**DOI:** 10.1186/1476-511X-13-94

**Published:** 2014-06-07

**Authors:** Shun Xu, Jie Cheng, Yu-ning Chen, Keshen Li, Ze-wei Ma, Jin-ming Cen, Xinguang Liu, Xi-li Yang, Can Chen, Xing-dong Xiong

**Affiliations:** 1 Institute of Aging Research, Guangdong Medical College, Dongguan, P.R. China; 2 Guangdong Provincial Key Laboratory of Medical Molecular Diagnostics, Dongguan, P.R. China; 3 Institute of Biochemistry & Molecular Biology, Guangdong Medical College, Zhanjiang, P.R. China; 4 Key Laboratory of Neurodegenerative Disease and Aging Research, Affiliated Hospital of Guangdong Medical College, Zhanjiang, P.R. China; 5 Department of Cardiovascular Disease, The First People’s Hospital of Foshan, Foshan, P.R. China; 6 Department of Cardiovascular Disease, The Affiliated Hospital of Guangdong Medical College, Zhanjiang, P.R. China

**Keywords:** *LRP6*, Single nucleotide polymorphism, Myocardial infarction, Risk

## Abstract

**Background:**

Abnormal lipids is one of the critical risk factors for myocardial infarction (MI), however the role of genetic variants in lipid metabolism-related genes on MI pathogenesis still requires further investigation. We herein genotyped three SNPs (*LRP6* rs2302685, *LDLRAP1* rs6687605, *SOAT1* rs13306731) in lipid metabolism-related genes, aimed to shed light on the influence of these SNPs on individual susceptibility to MI.

**Methods:**

Genotyping of the three SNPs (rs2302685, rs6687605 and rs13306731) was performed in 285 MI cases and 650 control subjects using polymerase chain reaction–ligation detection reaction (PCR–LDR) method. The association of these SNPs with MI and lipid profiles was performed with SPSS software.

**Results:**

Multivariate logistic regression analysis showed that C allele (OR = 1.62, *P* = 0.039) and the combined CT/CC genotype (OR = 1.67, *P* = 0.035) of *LRP6* rs2302685 were associated with increased MI risk, while the other two SNPs had no significant effect. Further stratified analysis uncovered a more evident association with MI risk among younger subjects (≤60 years old). Fascinatingly, CT/CC genotype of rs2302685 conferred increased LDL-C levels compared to TT genotype (3.0 mmol/L *vs* 2.72 mmol/L) in younger subjects.

**Conclusions:**

Our data provides the first evidence that *LRP6* rs2302685 polymorphism is associated with an increased risk of MI in Chinese subjects, and the association is more evident among younger individuals, which probably due to the elevated LDL-C levels.

## Background

Myocardial infarction (MI) is a leading cause of death and morbidity worldwide, which is a main manifestation of coronary artery disease (CAD). Previous studies and clinical trials have established multiple risk factors contributing to the pathogenesis of MI, including obesity, hypercholesterolemia, smoking, alcohol intake, diabetes, hypertension, physical inactivity and psychosocial situation [1-3]. Among these, hypercholesterolemia arising from abnormal lipid metabolism has been considered to be one of the most key risk factors for MI [4,5]. What’s more, apart from above modifiable risk factors, a growing body of studies have demonstrated close associations of genetic variants in candidate genes with the risk of MI, suggesting that host genetic backgrounds exert critical roles on MI pathogenesis as well [6-8].

Low density lipoprotein receptor-related protein 6 (*LRP6*), a member of the LRP family of type I transmembrane proteins, functions as a co-receptor with Frizzled proteins for Wnt ligands, and thus plays a critical role in the regulation of multiple cellular processes, and the development of many human diseases [9-11]. Moreover, accumulating evidences have recently linked *LRP6* genetically to early coronary artery disease and abnormal lipids including hypercholesterolemia [12-14]. Five functional mutations (K82N, S488Y, P1066T, P1206H and I1264V) within *LRP6* gene have been identified in CAD patients, which might be contributing factors for CAD through significantly reduction in both LRP6 protein level and Wnt signal activity [15]. Another mutation in *LRP6* (R611C) has been identified in an Iranian family characterized with early CAD, features of the metabolic syndrome (hyperlipidemia, hypertension and diabetes), and osteoporosis [16], which significantly promoted PDGF-dependent vascular smooth muscle cells (VSMCs) proliferation compared to wild-type *LRP6*[17]. Thus it was reasonable to speculate that *LRP6* might probably play an important role in MI pathogenesis.

Low density lipoprotein receptor adaptor protein 1 (*LDLRAP1*) interacts with the cytoplasmic tail of LDL receptor and exerts a crucial role on LDL uptake *via* promoting LDL receptor clustering into clathrin-coated pits [18-21]. In addition, mutations in *LDLRAP1* gene cause familial hypercholesterolemia (FH) characterized with severe hypercholesterolemia and premature coronary artery disease [22,23]. Sterol O-acyltransferase 1 (*SOAT1*) is also named acyl-coenzyme A: cholesterol acyltransferase (*ACAT*), which esterifies cholesterol in a variety of tissues [24]. Previous studies have demonstrated that *SOAT1* was involved in the formation of atherosclerotic plaques, and thus might be a promising target for atherosclerosis and hypercholesterolemia treatment [25,26]. In spite of the close association between these two genes and hypercholesterolemia, the effects of *LDLRAP1* and *SOAT1* polymorphisms on hypercholesterolemia and CAD remain largely unknown.

Single nucleotide polymorphism (SNP) has been established to influence individual susceptibility for numerous human diseases. A plethora of evidences have suggested that SNPs within the lipid metabolism-related genes might potentially contribute to MI risk [27-30]. Nonetheless, the genetic causes and underlying molecular mechanisms of these candidate genes for MI are still far to be elucidated. Thus, we herein conducted a case–control study to investigate the association of the three SNPs in the lipid metabolism-related genes (rs2302685 in *LRP6*, rs6687605 in *LDLRAP1* and rs13306731 in *SOAT1*) with the risk of MI. Our data revealed that the C allele of rs2302685 in *LRP6* has a significant association with an increased risk of MI in a Chinese population, which probably due to the elevated LDL-C levels.

## Methods

### Study subjects

285 MI patients and 650 control subjects were consecutively recruited from the First People’s Hospital of Foshan (Foshan, China) and the Affiliated Hospital of Guangdong Medical College (Zhanjiang, China) from March 2011 to February 2013. The diagnosis of MI was based on typical electrocardiographic changes and on increases in the serum cardiac markers, such as creatinine kinase, aspartate aminotransferase, lactate dehydrogenase and troponin T. The diagnosis was confirmed by the identification of the responsible stenosis in any of the major coronary arteries or in the left main trunk by coronary angiography. Subjects with a history of hematologic, neoplastic, renal, liver, or thyroid diseases were excluded. The unaffected controls were judged to be free of MI by questionnaires, medical history, clinical examination and electrocardiography.

All subjects enrolled in this study were genetically unrelated ethnic Han Chinese. Each subject was interviewed to collect information on demographic data and risk factors related to MI after obtaining the informed consent. The study was approved by the Medical Ethics Committee of the First People’s Hospital of Foshan and the Affiliated Hospital of Guangdong Medical College.

### Biochemical parameters analysis

The blood sample drawn from each subject was centrifuged at 2000 × g for 15 min immediately after collection and stored at −80°C. The levels of plasma total cholesterol (TC), triglyceride (TG), high density lipoprotein cholesterol (HDL-C), and low density lipoprotein cholesterol (LDL-C) were measured enzymatically using a chemistry analyzer (Olympus, Japan). Glucose was analyzed by the glucose oxidase method with an Abbott V/P Analyzer (Abbott Laboratories, USA).

### DNA extraction

Genomic DNA was extracted from peripheral whole blood by TIANamp blood DNA extraction kit (TianGen Biotech, Beijing, China) according to the manufacturer’s instructions. All DNA samples were dissolved in water and stored at −20°C until use.

### Genotyping

SNPs genotyping were performed utilizing polymerase chain reaction-ligase detection reaction (PCR-LDR) method (Shanghai Biowing Applied Biotechnology Company), as described in our previous study [31]. The sequence of primers and probes were listed in Additional file 1: Table S1.

### Statistical analysis

All the three SNPs were tested for confirmation with Hardy-Weinberg expectations by a goodness-of-fit χ^2^ test among the control subjects. Quantitative variables were expressed as mean ± standard deviation (SD), and qualitative variables were expressed as percentages. The differences of the demographic characteristics between the cases and controls were estimated using the χ^2^ test (for categorical variables) and Student’s *t* test (for continuous variables).

Multivariate association analyses with MI risk, genotype frequencies were assessed by means of multivariate methods based on logistic regression analysis, the odds ratios (ORs) and 95% confidence intervals (CIs) for the effect of SNPs on MI risk adjusted by age, sex, smoking, drinking, hypertension, diabetes and hyperlipidemia. Association analyses between SNPs and blood lipid profiles were performed by one-way analysis of variance (ANOVA). The statistical analyses were performed using the SPSS software (version 21). A *P* value of less than 0.05 was used as the criterion of statistical significance.

## Results

### Characteristics of the study population

The characteristics of MI cases and control subjects were listed in Table 1. No statistically significant difference between cases and controls was observed in terms of age. In the lipid profiles comparison, TG and LDL-C were significantly higher in MI patients than in controls (*P* < 0.001, *P* < 0.001, respectively), whereas serum HDL-C levels were significantly higher among controls (*P* < 0.001). Besides, the average fasting plasma glucose (FPG) in MI cases was significantly higher than that of the controls (*P* < 0.001). MI cases had higher levels of systolic blood pressure, diastolic blood pressure; the prevalence of smokers, alcohol consumers, and individuals with hypertension, diabetes or hyperlipidemia was significantly higher among the MI patients. In addition, the number of female subjects in MI cases was much lower than the male subjects. In all, these data demonstrated that male gender, smoking, alcohol intake, hypertension, hyperlipidemia and diabetes mellitus were the important risk factors for MI development in Chinese population.

**Table 1:** The characteristics of MI cases and controls

**Variable**	**Controls (n = 650)**	**Cases (n =285)**	***P*-value**^**a**^
Age (years)	61.61 ± 12.22	62.07 ± 11.99	0.591
Sex (male)	377 (58.0%)	221 (77.5%)	**<0.001**^**b**^
Smoking	168 (25.8%)	171 (60.0%)	**<0.001**
Drinking	94 (14.5%)	77 (27.0%)	**<0.001**
Hypertension	233 (35.8%)	179 (62.8%)	**<0.001**
Diabetes	105 (16.2%)	136 (47.7%)	**<0.001**
Hyperlipidemia	245 (37.7%)	201 (70.5%)	**<0.001**
Systolic BP (mm Hg)	132.53 ± 18.98	140.02 ± 19.16	**<0.001**
Diastolic BP (mm Hg)	72.86 ± 10.47	75.66 ± 11.56	**<0.001**
FPG (mmol/L)	5.81 ± 1.91	6.64 ± 1.72	**<0.001**
Triglycerides (mmol/L)	1.49 ± 0.82	2.06 ± 0.97	**<0.001**
Total cholesterol (mmol/L)	4.62 ± 1.16	4.71 ± 1.21	0.242
LDL cholesterol (mmol/L)	2.63 ± 0.92	3.03 ± 0.97	**<0.001**
HDL cholesterol (mmol/L)	1.37 ± 0.67	1.18 ± 0.36	**<0.001**

^a^Two-sided chi-square test or independent-samples *t*-test.

^b^*P* values under 0.05 were indicated in bold font.

### Multivariate associations of three SNPs with the risk of MI

Three SNPs (rs2302685 in *LRP6*, rs6687605 in *LDLRAP1* and rs13306731 in *SOAT1*) were genotyped in 285 MI patients and 650 control subjects. The primary information for rs2302685, rs6687605 and rs13306731 polymorphisms was listed in Table 2. Minor allele frequency (MAF) of all three SNPs in our controls was similar to MAF for Chinese in HapMap database (Table 2). All the genotype frequency distributions of the three SNPs in our control subjects followed Hardy-Weinberg equilibrium proportions (all *P* values ≥ 0.10, Table 2).

**Table 2:** Primary information for rs2302685, rs6687605 and rs13306731 SNPs

**Genotyped SNPs**	**rs2302685**	**rs6687605**	**rs13306731**
Chr Pos (Genome Build 104.0)	12301898	25889632	179320578
Gene	LRP6	LDLARP1	SOAT1
MAF^a^ for Chinese (CHB) in HapMap	0.138	0.476	0.354
MAF in our controls (n = 650)	0.064	0.419	0.291
*P* Value for HWE^b^ test in our controls	0.670	0.100	0.180

^a^MAF: minor allele frequency.

^b^HWE: Hardy–Weinberg equilibrium.

The allele and genotype distributions of the three SNPs in the cases and the controls were shown in Table 3. From the allelic association analysis, we found only rs2302685 showed statistical significance and C allele was associated with a significantly increased risk of MI (OR = 1.62, 95% CI = 1.03-2.55, *P* = 0.039, Table 3). In addition, the combined CT/CC genotype exhibited an increased risk of MI as well (OR = 1.67, 95% CI = 1.04-2.67, *P* = 0.035, Table 3), compared to TT genotype. These data indicated that *LRP6* SNP rs2302685 was associated with MI risk, and that individuals carrying C allele might have significantly increased MI susceptibility. However, we did not find any association between rs6687605 or rs13306731 and the risk of MI (Table 3).

**Table 3:** Multivariate associations of the SNPs with the risk of MI

**Type**	**Controls (n = 650)**	**Cases (n = 285)**	**OR (95% CI)**^**a**^	***P*-value**^**a**^
	**No. (%)**	**No. (%)**		
***LRP6 rs2302685***				
T	1217 (93.6)	524 (91.9)	1.00	-
C	83 (6.4)	46 (8.1)	1.62 (1.03-2.55)	**0.039**^**b**^
TT	569 (87.5)	240 (84.2)	1.00	-
CT + CC	81 (12.5)	45 (15.8)	1.67 (1.04-2.67)	**0.035**
***LDLRAP1 rs6687605***				
C	545 (41.9)	233 (40.9)	1.00	-
T	755 (58.1)	337 (59.1)	1.00 (0.78-1.27)	0.986
CC	104 (16.0)	46 (16.1)	1.00	-
CT + TT	546 (84.0)	239 (83.9)	1.04 (0.67-1.63)	0.854
***SOAT1 rs13306731***				
A	922 (70.9)	403 (70.7)	1.00	-
G	378 (29.1)	167 (29.3)	1.07 (0.83-1.39)	0.584
AA	334 (51.4)	142 (49.8)	1.00	-
GA + GG	316 (48.6)	143 (50.2)	1.04 (0.74-1.44)	0.84

^a^Adjusted for age, sex, smoking, drinking, hypertension, diabetes and hyperlipidemia.

^b^*P* values under 0.05 were indicated in bold font.

### Stratification analyses of *LRP6* rs2302685 polymorphism and risk of MI

We further evaluated the alleles or genotypes of *LRP6* rs2302685 and MI susceptibility after stratifying the subjects by age, sex, status of smoking or drinking. Stratification analyses by age (≤60 or > 60 years old) revealed that the increased risk of MI was more evident among younger subjects (≤60 years old) carrying C allele (Table 4, OR = 2.46, 95% CI = 1.20-5.03, *P* = 0.014) or the combined CT/CC genotype (Table 4, OR = 2.46, 95% CI = 1.19-5.06, *P* = 0.015), whereas no significant association was observed from the group older than 60 years old (Table 4). No more evident association between *LRP6* rs2302685 polymorphism and risk of MI was observed among subgroups by sex, status of smoking or drinking (data not shown).

**Table 4:** **Multivariate associations of the rs2302685 in *LRP6 *** **gene with the risk of MI by further stratification for age**

**Type**	**Controls no. (%)**	**Cases no. (%)**	**OR (95% CI)**^**a**^	***P*-value**^**a**^
**≤60 y**	**n = 297**	**n = 130**		
T	553 (93.1)	233 (89.6)	1.00	-
C	41 (6.9)	27 (10.4)	2.46 (1.20-5.03)	**0.014**^**b**^
TT	256 (86.2)	104 (80.0)	1.00	-
CT + CC	41 (13.8)	26 (20.0)	2.46 (1.19-5.06)	**0.015**
**> 60 y**	**n = 353**	**n = 155**		
T	664 (94.1)	291 (93.9)	1.00	-
C	42 (5.9)	19 (6.1)	1.16 (0.62-2.19)	0.636
TT	313 (88.7)	136 (87.7)	1.00	-
CT + CC	40 (11.3)	19 (12.3)	1.22 (0.63-2.35)	0.557

^a^Adjusted for sex, smoking, drinking, hypertension, diabetes and hyperlipidemia.

^b^*P* values under 0.05 were indicated in bold font.

### Association analysis between *LRP6* rs2302685 polymorphism and LDL-C levels

In order to probe into the potential explanation to the enhanced effects of *LRP6* rs2302685 polymorphism on MI risk among younger subjects (≤60 years old), we further analyzed the association between *LRP6* rs2302685 polymorphism and LDL-C, HDL-C, TC and TG levels. Though none of the above lipids profile exhibited significant association with *LRP6* rs2302685 polymorphism among total subjects (data not shown), CT/CC genotype of rs2302685 conferred 0.28 mmol/L increase in LDL-C levels compared to TT genotype (3.00 mmol/L *vs* 2.72 mmol/L*, P* = 0.047) in younger subjects, whereas no significant association was observed between rs2302685 and HDL-C, TC and TG levels (Table 5). Thus, the results indicated that the increased risk of *LRP6* rs2302685 polymorphism in MI was more evident among younger subjects might be probably due to the elevated LDL-C levels.

**Table 5:** ANOVA analysis of the association between rs2302685 in LRP6 gene and the LDL-C, HDL-C, TC and TG levels by further stratification for age

**Variable**	**≤60**			**> 60**		
	**TT**	**CT + CC**	***P*-value**^**a**^	**TT**	**CT + CC**	***P*-value**^**a**^
LDL cholesterol (mmol/L)	2.72 ± 1.00	3.00 ± 1.04	**0.047**^**b**^	2.76 ± 0.91	2.63 ± 0.86	0.323
HDL cholesterol (mmol/L)	1.36 ± 0.83	1.31 ± 0.40	0.658	1.28 ± 0.39	1.29 ± 0.36	0.831
Total cholesterol (mmol/L)	4.65 ± 1.18	4.93 ± 1.24	0.077	4.63 ± 1.17	4.37 ± 1.04	0.103
Triglycerides (mmol/L)	1.79 ± 1.04	1.68 ± 1.00	0.415	1.57 ± 0.77	1.56 ± 0.73	0.965

^a^Two-sided chi-square test or independent-samples *t*-test.

^b^*P* values under 0.05 were indicated in bold font.

## Discussion

The principal pathogenesis of MI is the disruption of coronary atherosclerotic plaques [32], which caused by both individual’s genetic makeup and various environmental factors. Previous studies have demonstrated the effects of *LRP6* in early coronary artery disease and abnormal blood lipids including hypercholesterolemia [12-14], indicating the important role of *LRP6* in the MI development. Nonetheless, the association between SNPs in *LRP6* gene and MI risk is still largely unknown. In this study, we performed a genetic association analysis on the three SNPs (rs2302685 in *LRP6*, rs6687605 in *LDLRAP1* and rs13306731 in *SOAT1*), and revealed that the *LRP6* rs2302685 polymorphism was associated with increased risk of MI in a Chinese Han population, and the association was more remarkable among younger subjects (≤60 years old), which might potentially due to the enhanced LDL-C levels. Taken together, our study suggested that *LRP6* rs2302685 might play an important role in the MI pathogenesis.

Though rs2302685 has been considered as a common functional *LRP6* polymorphism, and is significantly associated with several human diseases such as Alzheimer’s disease [33], the effects of this polymorphism on MI risk is still unknown. Nonetheless, Sarzani *et al.* has reported that the rs2302685 was strongly related to carotid artery atherosclerosis (CAA) in hypertensive patients, indicating that C allele of *LRP6* rs2302685 might be an independent risk factor for CAA (OR = 2.08, 95% CI = 1.27-3.41, *P* = 0.003) [34]. Carotid artery atherosclerosis is closely associated with arterial cardiovascular events, and is a strong predictor of future myocardial infarction, which might share common risk factors [35,36]. Our data that rs2302685 endowed C allele carriers with significant increased MI risk was in consistent with the results from the above association analysis between rs2302685 and CAA.

Our stratified analyses revealed that the increased risk of *LRP6* rs2302685 polymorphism in MI was more evident among younger subjects (≤60 years old), whereas no significant association was observed from the group older than 60 years old (Table 4). In addition, *LRP6* rs2302685 only exhibited an association with elevated LDL-C levels in younger individuals, but not in total or older subjects. Weak immune system and relative high level exposure to environmental risk factors in older individuals may account for these. The potential risk of MI in older subjects is more likely due to the aging effects rather than direct genetic effects. Thus, the *LRP6* rs2302685 polymorphism might be more influential in early-onset MI, which was similar as the effects of *LRP6* R611C variant on early-onset CAD development in an Iranian family [16].

Previous investigations have demonstrated that LRP6, as a component of LDL cholesterol trafficking complex, was involved in direct LDL uptake [37]; and the elevated LDL-C levels in *LRP6* R611C mutation carriers was likely due to the reduced LDL clearance capacity [16]. Moreover, Tomaszewsk *et al.* unveiled that T allele of *LRP6* rs10845493 polymorphism was associated with 0.14 mmol/L increase in LDL-C levels (SE = 0. 05, *P* = 0.0038) [38]. In consistent with above findings, our data revealed that the LDL-C levels of the individuals carrying CT/CC genotype were higher than the TT genotype carriers among younger individuals (≤60 years old) (Table 5), which provided a reasonable explanation to the enhanced effects of rs2302685 on MI pathogenesis in younger subjects.

Several limitations herein in this case–control study need to be addressed. First, the case subjects and controls enrolled from hospitals may not represent the general population. Nonetheless, the genotype distribution of the controls was in Hardy-Weinberg equilibrium. Second, the moderate sample size of our study limited the statistical power, especially in the case subjects. Finally, further studies in different population could help to verify the true significance of the association between the rs2302685 polymorphism and the risk of MI. However, our observations provided valuable insights and interesting information and might serve to guide future studies in this area.

## Conclusions

In aggregate, our study firstly unveiled that the C allele of *LRP6* rs2302685 was associated with an increased risk of MI in a Chinese population, and the association was more evident among younger subjects, which potentially due to the elevated LDL-C levels. Further studies with larger sample size and in diverse ethnic populations are required to confirm the general validity of our findings.

## Abbreviations

*LRP6*: Low density lipoprotein receptor-related protein 6; MI: Myocardial infarction; SNP: Single nucleotide polymorphism; CAD: Coronary artery disease; PCR-LDR: Polymerase chain reaction-ligase detection reaction; OR: Odds ratio; CI: Confidence interval; LDL-C: Low density lipoprotein cholesterol; TC: Total cholesterol; TG: triglyceride; HDL-C: High density lipoprotein cholesterol.

## Competing interests

The authors declare no competing interests.

## Authors’ contributions

SX, JC and Z-wM carried out the molecular genetic studies and the statistical analysis, and SX drafted the manuscript. Y-nC and JC carried out the genotyping. J-mC, X-lY and CC helped to collect study subjects. SX and X-dX participated in the design of the study. KL, XL and X-dX helped to revise the manuscript. All authors read and approved the final Manuscript.

## Supplementary Material

Additional file 1: Table S1 The sequences of the primers and probes used to genotype the SNPs. Click here for file
