# Supplementary material for: The LRP6 rs2302685 polymorphism is associated with increased risk of myocardial infarction
Source: Lipids Health Dis. 2014 Jun 7;13:94. doi: 10.1186/1476-511X-13-94 (PMC4059096; doi:10.1186/1476-511X-13-94)
Supplement: Additional file 1: Table S1 — The sequences of the primers and probes used to genotype the SNPs. [file 1476-511X-13-94-S1.doc]

**Table S1 The sequences of the primers and probes used to genotype the SNPs**

| **Name** | **Sequence (5’-3’)** |
| --- | --- |
| **Primers** |  |
| rs2302685-F | GTAACATACCCTTTCTCTGG |
| rs2302685-R | AGATCAGTTGGAGTGGTGC |
| rs6687605-F | AAAGCCAGCCAAGAGGGAG |
| rs6687605-R | CGTCAGGACTCACAGCTCT |
| rs13306731-F | CTTGGGCAATGGAGTCTTAC |
| rs13306731-R | GACTCACATTTTTCAGAGG |
| **Probes** |  |
| rs2302685-FAM | P-TTGTGGTAAACCCAGAGAAAGGGTATTTTTTTTTTTTTTTTTTTTTTTTTTTTTTTTTTTTTTTTTTTTTTTTTTTTTT-FAM |
| rs2302685-C | TTTTTTTTTTTTTTTTTTTTTTTTTTTTTTTTTTTTTTTTTTTTTTTTTTTTTTTTTTTTTTTTTTTTTTTTTTTTTTGGCGAGCAGGACAGACCTCGAGCCG |
| rs2302685-T | TTTTTTTTTTTTTTTTTTTTTTTTTTTTTTTTTTTTTTTTTTTTTTTTTTTTTTTTTTTTTTTTTTTTTTTTTTTTTTTTGGCGAGCAGGACAGACCTCGAGCCA |
| rs6687605-FAM | P-GGGGGTGCAGTCTTGGCGGGTTTTTTTTTTTTTTTTTTTTTTTTTTTTTTTT-FAM |
| rs6687605-C | TTTTTTTTTTTTTTTTTTTTTTTTTTTTTTCAGGACTCACAGCTCTTCAAGGG |
| rs6687605-T | TTTTTTTTTTTTTTTTTTTTTTTTTTTTTTTTCAGGACTCACAGCTCTTCAAGGA |
| rs13306731-FAM | P-GCACTGTCCTCTGAAAAATGTGAGTTTTTTTTTTTTTTTTTTTTTTTTTTTTTTTTTTTTTTTTTTTTTTTTTTTTTTT-FAM |
| rs13306731-G | TTTTTTTTTTTTTTTTTTTTTTTTTTTTTTTTTTTTTTTTTTTTTTTTTTTTTTTTTTATTCTCAAGAATGGTATGCACGTCG |
| rs13306731-A | TTTTTTTTTTTTTTTTTTTTTTTTTTTTTTTTTTTTTTTTTTTTTTTTTTTTTTTTTTTTATTCTCAAGAATGGTATGCACGTCA |
